# Advanced Early-Onset Fahr’s Disease: A Case Report

**DOI:** 10.7759/cureus.39495

**Published:** 2023-05-25

**Authors:** Kristopher Aghemo, Ryan Salmanzadeh, Osmany DeAngelo, Austin M Salmanzadeh

**Affiliations:** 1 Radiology, Larkin Community Hospital, Miami, USA; 2 Biology, University of Central Florida, Orlando, USA

**Keywords:** movement disorders, basal ganglia calcification, striopallidodentate calcinosis, microcephaly, fahr’s syndrome, fahr’s disease

## Abstract

Fahr’s disease is a rare disorder characterized by abnormal calcium deposition within the basal ganglia, cerebellar dentate nuclei, and white matter tracts with subsequent atrophy. Typical CT imaging features include extensive symmetric calcification involving the basal ganglia and subcortical white matter. Primary Fahr’s disease (also known as primary familial brain calcification) is diagnosed based on the exclusion of secondary causes such as underlying metabolic or endocrine disorders. The disease may or may not feature a detectable genetic component, which is inherited in an autosomal dominant or recessive pattern. Fahr’s disease typically presents in the fourth to fifth decade of life and often manifests clinically with movement disorders and/or neuropsychiatric symptoms ranging from memory/concentration deficits to psychosis. Fahr’s disease is not fully understood and is often misdiagnosed in psychiatric patients, thus further literature and documentation of characteristic imaging findings would prove helpful when the diagnosis is suspected. We demonstrate a very radiologically advanced case of Fahr’s disease particularly in terms of calcifications in a contrastingly young patient with atypical clinical findings of gait abnormalities, microcephaly, and schizophrenia. Although genetic testing and family history were unavailable for this patient, the profound imaging and symptom presentations should serve to expand the awareness and understanding of a Fahr's disease diagnosis in younger and older patients alike.

## Introduction

Fahr’s disease (Striopallidodentate calcinosis) was first documented in 1930 by Karl Theodore Fahr and has since been defined as symmetric bilateral basal ganglia calcification in the absence of an explainable underlying abnormality such as endocrine disorders, biochemical disorders, infections, toxins, or history of trauma [1]. It is classified as a neurodegenerative disease typically manifesting from ages 40-60 years old and often found incidentally on CT imaging in patients who present with non-specific symptoms such as movement disorders, seizures, gait abnormalities, dementia, or cognitive deficits [2]. Brain calcification might allude to an increase in ischemic stroke probability, however, at the time of writing, only two case reports exist that demonstrate stroke as a symptom of Fahr’s disease. The diagnosis is made based on positive imaging findings in the setting of neuropsychiatric or movement disorders while excluding coexisting diseases or disorders previously mentioned that could otherwise explain the calcifications [2]. If calcifications can be attributed to one or more of the aforementioned disorders, the diagnosis must be classified as “Fahr’s syndrome” rather than “Fahr’s disease” [2].

Although the pathogenesis of the calcifications is not completely understood, rudimentary theories exist based on advanced imaging, histological studies, and knowledge of other basal ganglia deposition diseases. There is currently no curative treatment for Fahr’s disease, therefore, treatment is supportive and focused on improving neuropsychiatric symptoms and seizure prophylaxis. We demonstrate a case of Fahr’s disease in a 38-year-old man who presented with generalized weakness and psychiatric symptoms. He was incidentally found to have extensive bilateral basal ganglia, thalami, dentate nuclei, and white matter tract calcifications in the setting of normal lab values.

## Case presentation

A 38-year-old male with a history of schizophrenia presented with two days of generalized weakness and staggered and stiff movement. The physical exam was unremarkable aside from microcephaly. Neurologic examination revealed cognitive deficits in attention/memory, orientation to only person and place, as well as a spastic ataxic gait. Laboratory analysis, including a hemogram screen, was unremarkable, vital organ function was normal as were calcium, magnesium, and phosphorous levels. Testing for infectious diseases such as toxoplasmosis was also found to be inconclusive. Collateral information was not available.

**Figure 1:**
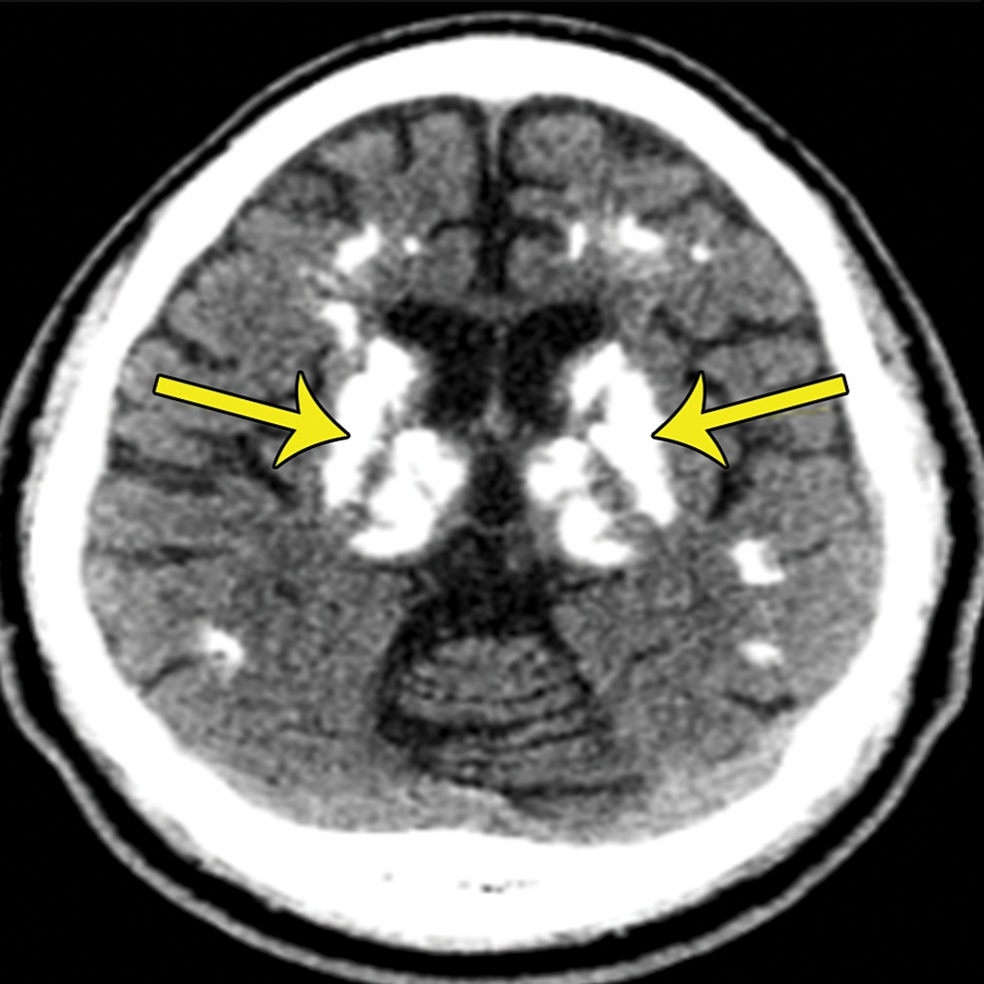
The axial non-contrast CT scan demonstrates symmetric calcification of the thalami and basal ganglia (yellow arrows) CT: computed tomography

**Figure 2:**
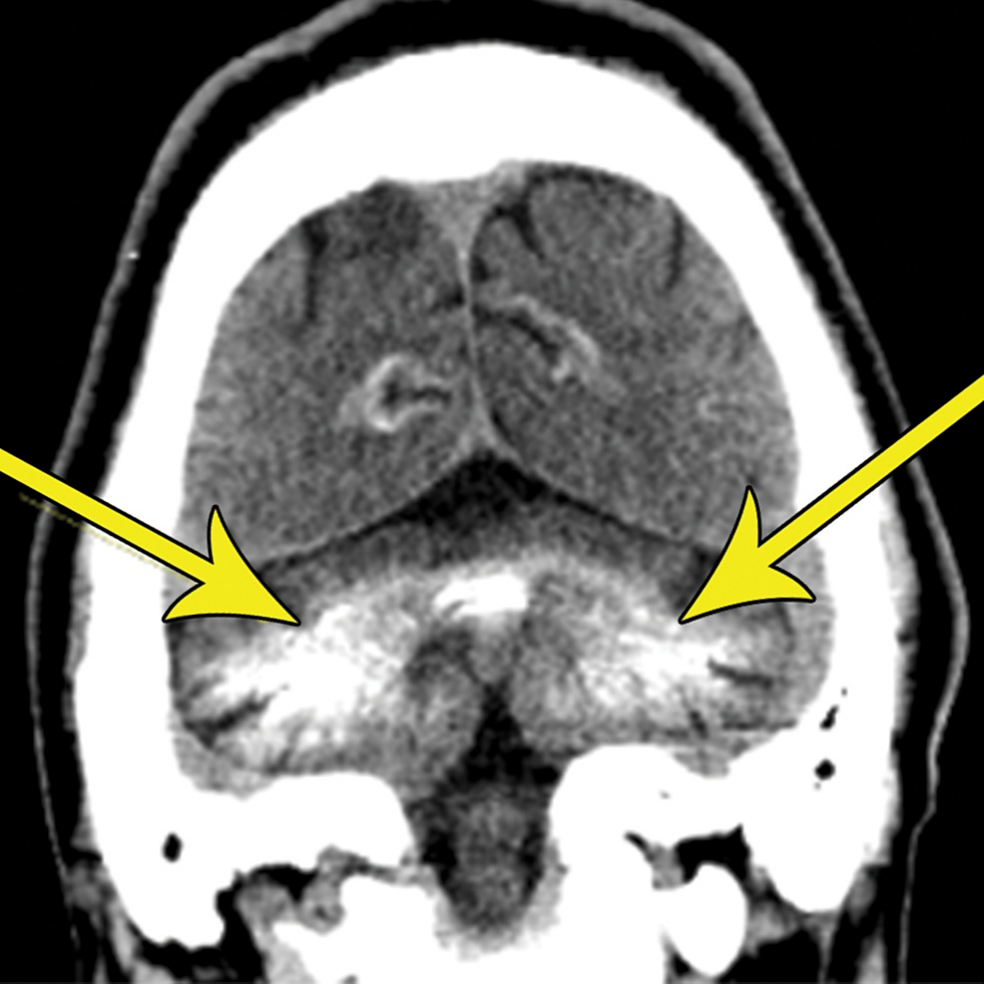
The coronal non-contrast CT scan demonstrates symmetric calcification of the cerebellum and dentate nuclei (yellow arrows) CT: computed tomography

**Figure 3:**
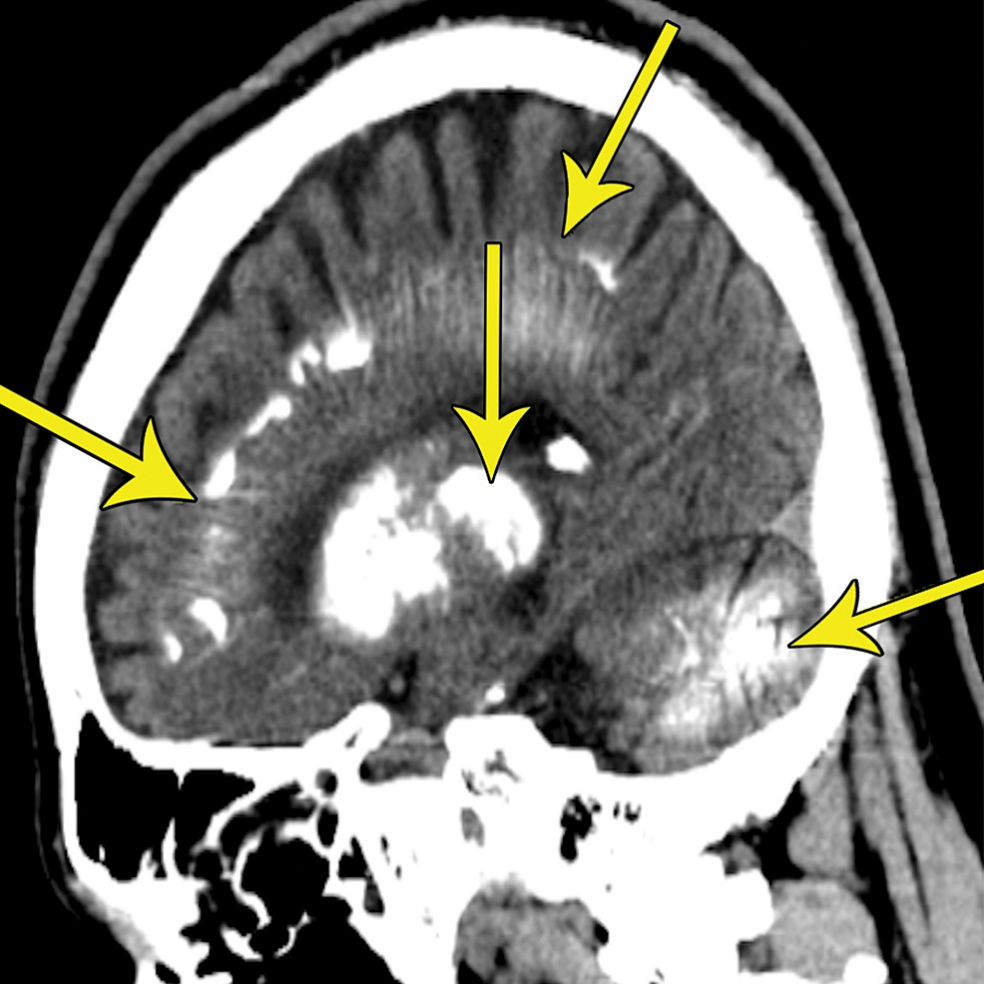
Sagittal non-contrast CT demonstrates extensive symmetric calcification of the subcortical gray-white junctions, basal ganglia, thalami, cerebellar nuclei, and corona radiata (yellow arrows) CT: computed tomography

Evaluation with a non-contrast CT scan of the head (Figures 1-3) revealed extensive calcification of the cerebral hemispheres, dentate nuclei, and basal ganglia as well as bilateral frontal subcortical and corticomedullary calcifications.

Fahr’s disease was suspected based on imaging and clinical presentation. The diagnosis of Fahr’s disease was further supported after excluding secondary etiologies and other diseases that also feature basal ganglia calcifications.

An early onset variant of Fahr’s disease typically occurs in individuals aged 40 or less in which affected patients first develop psychiatric symptoms that are later followed by movement disorders. The patient evaluated in this case in his late 30s presented with a symptom timeline consistent with this variant: the history of schizophrenia followed by the gait abnormalities for which the patient was initially evaluated.

Currently, only symptomatic treatment is available for movement and psychiatric disorders, as there is no cure for the calcifications caused by Fahr's disease. Typical treatments for Parkinsonian movement symptoms, such as levodopa, have proved mostly ineffective [3]. It should be noted that typical or first-generation antipsychotics should be avoided, as they tend to exacerbate pyramidal symptoms and increase susceptibility to neuroleptic malignant syndrome. Therefore, atypical or second-generation antipsychotics were considered instead. As Fahr’s disease is a neurodegenerative and progressive condition, the progression of this case can only be measured through longitudinal CT scanning over the course of the patient's life. It can be assumed the initial movement and psychiatric symptoms will only increase in severity over time as more of the corresponding areas of the basal ganglia and frontal cortex are further atrophied.

## Discussion

Fahr’s disease is highly variable in presentation, ranging from zero symptoms to a combination of severe movement and neuropsychiatric disease [4]. In a study analyzing symptom involvement in 99 cases of Fahr’s disease, 33% of cases were symptomatic [4]. Among the symptomatic patients, 55% exhibited movement disorders, followed by 39% with cognitive deficits, 36% with speech impairments, 36% with cerebellar deficits, and 31% featuring neuropsychiatric disorders such as schizophrenia [4]. Disorders involving gait abnormalities were found to be exceedingly rare, although our case did feature a spastic, ataxic gait [5].

Two distinct clinical variants of Fahr’s disease have been proposed by other studies: a variant of early onset (age<40) and late-onset (age >50) [6]. In the early variant, psychiatric symptoms were found to present earlier, before movement disorders. This was consistent with our patient who was in his late 30s with a history of schizophrenia-like symptoms, presenting with new-onset gait abnormalities and generalized weakness [6]. Our patient’s new-onset movement disorder is likely indicative of a level of calcification that eclipsed a certain threshold causing his symptoms. In the late-onset variant, the opposite presentation of symptoms is usually observed, which is an initial presentation with severe movement disorder followed by dementia-like symptoms [6].

Pathophysiology

The pathophysiology of Fahr’s disease is not yet fully understood. Current theories are based on processes that involve calcifications of the basal ganglia. These theories may be categorized into three groups: 1. Anatomical anomalies, 2. Vascular membrane defects, 3. Dysregulation of metabolic and/or inflammatory processes [2,4,5,7].

Anatomic anomalies, such as a tortuous or narrowed arterial system supplying the basal ganglia, could be a cause of low blood flow and, subsequently, calcifications of the dependent structures. Advanced cerebral perfusion imaging studies in some cases have demonstrated severely impaired blood flow to areas of calcifications such as the basal ganglia and cerebral cortex, which may also explain the pathophysiology of the extrapyramidal symptoms [5]. Advanced positron emission tomography (PET) imaging studies have demonstrated decreased metabolism of glucose in both the basal ganglia and the frontal cortex in some patients with Fahr’s disease, which further supports the hypothesis of hypoperfusion [4]. The decreased glucose metabolism in the frontal cortex may also account for neuropsychiatric/behavioral abnormalities. 

Astrocyte necrosis surrounding areas of calcifications has been observed in histological studies. Within the affected area, mucopolysaccharide, calcium, and phosphorus depositions were identified. The extravasation of such substances may be attributed to defects in the vascular membrane, which has been demonstrated in some MRI studies [7].

The last theory, involving metabolic/inflammatory dysregulation, can be seen with other disease processes that tend to feature the deposition of substances within the basal ganglia. A few examples include neonatal hyperbilirubinemia, carbon monoxide poisoning, Wilson's disease, and hepatic encephalopathy among others. Undetectable pathologic processes involving the metabolism of certain compounds or subtle inflammatory processes, which lead to increased deposition of substances in the basal ganglia system have also been considered [2].

In regards to genetic patterns in Fahr’s disease, three distinct patterns have been identified: autosomal dominant, familial, and sporadic, with the names being self-explanatory [8]. The genetic pattern in our case remains unknown, as genetic testing was unavailable, and the patient was unable to give collateral information.

Diagnosis/imaging

The most sensitive modality in detecting Fahr’s disease is a non-contrast CT scan (NCCT), used in this case, which is better at detecting areas of calcifications compared to T2-weighted MRI [6]. However, CT findings do not often correlate with clinical features [7]. According to some studies, NCCT is superior in displaying areas of calcification compared to MRI but lacks a consistent correlation to clinical severity [8]. On the other hand, T2-weighted and fluid-attenuated inversion recovery (FLAIR) MRI studies in these patients understated the degree of calcifications while demonstrating hyperintensities in gray and white matter areas of the brain that were not calcified [8]. Interestingly, the hyperintensities of calcium calcification seen on MRI positively correlated with clinical severity [8]. The most involved area of hyperintensity was the bilateral white matter of the centrum semiovale, which positively correlated with dementia [8]. The MRI findings reinforce the theory that the disease process resulting in clinical symptoms is not limited to the calcification of structures and that there may be additional mechanisms not yet understood that result in clinical symptoms.

Diagnosing Fahr’s disease with NCCT requires the presence of bilateral, dense, symmetric calcific foci within the basal ganglia with a target measurement of at least 800 mm^2^ [9]. Other frequently involved areas include the thalamus, dentate nuclei, and deep cerebral white matter [7]. Since the imaging findings in Fahr’s disease are not specific, it is important for the clinician to rule out other differential diagnoses that may also present with bilateral calcifications of the basal ganglia. Differential diagnoses include poisoning with toxic compounds (CO, methanol, cyanide), biochemical diseases (hepatic encephalopathy or Wilson disease), other neurodegenerative diseases (Huntington's disease, Aicardi-Goutieres), infectious etiologies (toxoplasmosis), vascular pathologies (hypertensive infarcts or embolic events), and malignant neoplasms (primary central nervous system lymphoma) [7].

Differentiating Fahr’s disease from secondary etiologies and aforementioned differential diagnoses may be accomplished by obtaining normal lab values of calcium, phosphorus, parathyroid hormones, and other biochemical/infectious markers that, if positive, exclude Fahr’s disease.

## Conclusions

Fahr’s disease is a relatively rare pathology that features distinct imaging findings of bilateral calcifications in the basal ganglia and surrounding structures. It is often discovered via imaging, as a majority of these patients initially present subclinically. This disease is often mistaken for primary psychiatric and/or movement disorders resulting in inappropriate, and often ineffective, treatment. Therefore, it is important for clinicians to be aware of Fahr’s disease when evaluating patients with neuropsychiatric symptomatology. While we have an idea of how to differentiate the disease from other mimicking diagnoses, such as Parkinson’s disease or others, by methods of exclusion, there is still much to be learned regarding the pathophysiology of the disease. With further research, perhaps clinicians will someday be able to treat the underlying mechanism rather than the resulting symptoms. This case further expands the current understanding of Fahr’s disease, documenting some atypical findings, such as advanced disease in an early onset pattern, as well as microcephaly, while showcasing non-contrast CT findings in a detailed characteristic manner.
